# Exploring the Safewards Programme to Reduce Restrictive Practices in Residential Aged Care: Protocol for a Pilot and Feasibility Study

**DOI:** 10.1111/hex.70037

**Published:** 2024-09-30

**Authors:** Suzanne Dawson, Candice Oster, Michael Page, Stacey George

**Affiliations:** ^1^ Caring Futures Institute Flinders University Adelaide South Australia USA; ^2^ Uniting Communities Adelaide South Australia USA

**Keywords:** implementation, long‐term care, nursing homes, residential aged care, restraint, restrictive practices

## Abstract

**Introduction:**

Restrictive practice use in residential aged care homes internationally is unacceptably high. Although policies and legislation mandate the reduction or elimination of restrictive practices, there remains a gap in knowledge regarding strategies that have been effective in achieving a sustained reduction in restraint use. There is an urgent need to identify effective and feasible interventions that aged care staff can implement in everyday practice to reduce restraint use. Safewards is an evidence‐based programme that has demonstrated effectiveness in reducing conflict and restrictive practice use in inpatient psychiatric settings and has the potential to address the issue of restraint use in aged care homes. This study aims to evaluate the feasibility of Safewards in reducing restrictive practices in residential aged care homes.

**Methods:**

This pilot and feasibility study will adopt a mixed methods process and outcomes evaluation. Safewards will be implemented in two Australian residential aged care homes. The Reach, Effectiveness, Adoption, Implementation and Maintenance framework will be used to evaluate implementation outcomes. Additionally, the Consolidated Framework for Implementation Research will be used to guide qualitative data collection (including semi‐structured interviews with residents/family members, aged care leaders and staff) and explain the facilitators and barriers to effective implementation.

**Conclusion:**

This study will provide pilot evidence on the feasibility of the Safewards programme in residential aged care homes. Understanding the processes and adaptations for implementing and evaluating Safewards in residential aged care will inform a future trial in aged care to assess its effectiveness. More broadly, the findings will support the implementation of an international aged care policy of reducing restrictive practices in residential aged care.

**Patient or Public Contribution:**

A person with lived experience of caring for someone with dementia is employed as a Safewards facilitator and is a member of the steering committee. Residents and family members will be invited to participate in the project steering committee and provide feedback on their experience of Safewards.

**Trial Registration:**

ACTRN12624000044527.

## 1 Introduction

Restrictive practices are any practices or interventions that has the effect of restricting a person's freedom of movement or ability to make decisions [1]. Restrictive practices, which may include chemical, physical, environmental and/or mechanical restraint, as well as seclusion, must only be used as a last resort and in the least restrictive form. Restrictive practice use in residential aged care (RAC) homes internationally is unacceptably high [2]. The findings from the recent Royal Commission Into Aged Care Quality and Safety (2021) in Australia demonstrate that the range of solutions currently used in aged care has not reduced the need for or use of restrictive practices [3]. The Commission has highlighted the inappropriate use of restrictive practices as a form of abuse and a priority area to address [3]. Consequently, there is an urgent need for solutions specifically tailored to RAC homes.

The Safewards programme, an evidence‐based model and set of interventions developed to reduce restrictive practices in inpatient psychiatric settings [4], offers a possible solution. Safewards has been adapted for use in a range of settings where restrictive practices are used, including emergency departments and general medical wards. This study aims to determine the feasibility of implementing Safewards in RAC homes. Approximately 191,000 people are living in RAC in Australia, with most people presenting with high care needs for cognition and behaviour (81% people living with dementia and 48% for people without dementia) [5]. Cognitive decline and changes in behaviour can result in a range of safety concerns for residents and staff. Clinical guidelines state that nonpharmacological interventions should be prioritised as the first‐option interventions for people living with behavioural and psychological symptoms of dementia [6]. However, staff in RAC report the need for restrictive practices to ensure the safety of the person, other residents, staff and visitors and to prevent falls [7, 8]. A recent scoping review of the international literature reported that 30.7%–64.8% of people living with dementia in aged care have experienced restraint use [9]. However, restrictive practices cause significant harm, including death [10]. Consequences of restraints for individuals can be physical (e.g., pressure injuries, incontinence, falls and reduced mobility) and psychosocial (e.g., anger, depression, reduced independence and social isolation) [11, 12]. Furthermore, the use of restraints can negatively impact carers' and workers' well‐being [11].

In Australia, despite policy commitment, the acknowledged harms and multiple reviews recommending reduced reliance on restraints, restrictive practice remains prevalent in aged care homes [3]. Quality indicators for RAC in Australia report that the current rate of restraint use (including physical, mechanical and environmental restraint and seclusion) is 18.1% (a decrease of 23% from 12 months earlier) [13]. Research into restrictive practices in aged care homes shows that restraint use is influenced by a range of factors, including legislation and policy, and resident, staff and job characteristics [11]. The overuse of restraints in aged care has been found to be related to systemic issues rather than clinical needs, specifically inadequate staffing (skill mix and levels) and training [3, 8]. Proposals for reducing restrictive practice use include addressing attitudes and culture, enhancing training and education and reforming legislation and policies [14]. Several recent systematic reviews have examined interventions aimed at reducing physical restraint use in RAC [15, 16, 17]. Overall, there appears to be more evidence for multicomponent interventions (e.g., policy changes and education) rather than education alone [15, 16]. Strategies/interventions developed specifically to reduce overprescribing of psychotropic medication (a common restrictive practice) in RAC are also often multicomponent [18]. Previous studies have focused on training nursing staff and rarely include the voices of residents [15, 16]. To date, in Australia, reforms aimed at minimising restraint use have focused on amendments to legislation and policy [19, 20]. For example, aged care providers must monitor, review and report restraint use. Although providers are required to reduce restrictive practice use, there remains a gap in knowledge about the most effective strategies/interventions to achieve sustained reduction in restraint use in RAC. This study will extend current knowledge by testing a programme aimed at reducing conflict and restraint use. Training will be provided to personal care workers, who are the majority workforce in RAC in Australia, and residents will be included in the evaluation.

The reduction and elimination of restrictive practices has been a policy and practice focus in adult mental health inpatient services for the past two decades, providing a basis for examining best practices that may translate into aged care homes. Many strategies and interventions have been implemented to reduce the use of restrictive practices in a range of mental health care settings. However, these have often been ad hoc and without empirical evidence or a theoretical basis [21]. A recent review examining nonpharmacological interventions (*n* = 109) to reduce restrictive practices in adult mental health inpatient settings found that interventions were often poorly described, involved multiple procedures and were delivered in multiple ways [21]. Safewards is the most rigorously evaluated programme to reduce restraint use [4, 22, 23, 24] and stands out in the field of nonpharmacological interventions as a viable option for aged care.

Safewards, developed from an extensive research programme, includes a conceptual model that explains conflict and containment (restrictive practices) in acute psychiatric settings and a set of 10 interventions to reduce the rates of conflict and containment [4, 25, 26]. Safewards interventions address a broad range of factors that contribute to restrictive practice use in aged care homes, including staff characteristics (e.g., staff concerns about safety, lack of knowledge regarding the effects of restraint use and alternate practices, paternalistic attitudes and communication issues), patient characteristics (e.g., symptoms and demography), the physical environment and regulatory frameworks [1, 27]. A cluster randomised control trial conducted across 31 acute adult psychiatric units in the UK to evaluate the implementation of Safewards interventions reported reductions in conflict and the use of seclusion [4]. Safewards has been implemented broadly across multiple countries and settings [22], demonstrating the ability to successfully adapt the programme to different contexts; however, it has not yet been systematically tested in aged care homes.

In Australia, Safewards has been implemented in two states by the Departments of Health in Victoria and the ACT, with trials extending beyond adult mental health units to older person's mental health units, a general medical ward, an emergency department and an aged care residential setting [28]. All programme evaluations indicated that the implementation of Safewards was effective [28] in terms of improving seclusion rates, with the exception of aged care psychiatric units which were excluded from the analysis due to reporting zero or near zero seclusion events [29]. The recommendation from the Victorian Safewards trial in an aged care setting was that future evaluation focuses on changes in restraint use rather than seclusion rates, which is the more common practice in aged care [29]. Staff reports regarding the acceptability of the model and interventions were highly favourable, demonstrating that Safewards is an acceptable practice change intervention [28, 30]. Although Safewards has proven to be an effective programme in psychiatric settings, it is not known if it will be either effective or feasible to implement in RAC. To support and evaluate implementation, this study is informed by implementation frameworks.

Implementation science has developed to address the challenges in uptake of complex interventions in health care and other settings [31]. To date, however, there has been limited application of implementation theory and frameworks in aged care research [32]. Implementation science is a constantly evolving field with a large choice of theories, frameworks and models, which adds to the complexity regarding selection and reporting [33, 34, 35]. Three overarching aims of the use of theories, models and frameworks in implementation science have been identified as follows: (1) describing/guiding the process of implementation; (2) understanding/explaining what influences implementation and (3) evaluating implementation [31]. The updated Medical Research Council guidance for developing and evaluating complex interventions highlights the shift in focus from intervention effectiveness to understanding ‘whether and how the intervention will be acceptable, implementable, cost‐effective, scalable, and transferable across contexts’ [36, p. 2]. This project will implement Safewards in two RAC homes and evaluate the feasibility in these settings. Various learnings and recommendations from the Victorian Safewards trials will be applied to this project, including effective implementation strategies and evaluation recommendations [28, 37, 38].

### 1.1 Research Aim

To evaluate the feasibility of Safewards in reducing restrictive practices in RAC homes.

### 1.2 Research Questions

The research questions are as follows:

1. What is the Reach, Effectiveness, Adoption, Implementation and Maintenance (RE‐AIM) of Safewards in RAC?
2. What are the barriers and facilitators of implementing Safewards in RAC?
3. What are the recommendations for a future trial in RAC homes?

## 2 Methods

### 2.1 Study Design

This pilot study will utilise a mixed methods process and outcomes evaluation. Study findings will be reported in accordance with the CONSORT 2010 extension for pilot and feasibility trials [39]. The findings will be used to inform a future trial to assess the effectiveness of Safewards in reducing restrictive practices in aged care homes [40].

This study was registered as a trial with the Australian and New Zealand Clinical Trials Registry (registration no. ACTRN12624000044527).

### 2.2 Theoretical Frameworks

The RE‐AIM and Consolidated Framework for Implementation Research (CFIR) frameworks will be used to guide the study design and evaluation [41, 42, 43]. The RE‐AIM is an evaluation framework that describes the impact of the intervention alongside key issues that may facilitate or hinder broader impact [41]. The RE‐AIM describes outcomes according to Reach, Effectiveness, Adoption, Implementation and Maintenance [41]. The CFIR is a determinant framework that seeks to understand what influences implementation outcomes [31, 42, 43]. The CFIR considers five domains known to affect the process of implementation: Intervention Characteristics, Outer Setting, Inner Setting, Characteristics of Individuals and Process. It also includes 48 constructs that further elucidate factors influencing implementation outcomes [42, 43]. The multilevel domains (individual adopters, organisational and policy factors) acknowledge the multiple interacting influences [31, 42, 43]. Although determinant frameworks do not typically describe how change occurs, the recently updated CFIR has added the COM‐B constructs that describe how the individuals' *Capability* (e.g., skills), *Opportunity* (e.g., scope of practice/power) and *Motivation* (e.g., commitment to the intervention) influence behaviour change [43].

There is significant variation in the application of these theoretical frameworks. For example, the CFIR and RE‐AIM can be used to guide planning and/or evaluation [41, 42]. In this study, these frameworks will be used to guide the process evaluation, which will occur concurrently with the implementation but will not specifically change the implementation process. Currently, the RE‐AIM and CFIR are being used in combination across a range of implementation studies [44, 45, 46] that seek to determine the impact of interventions and strategies for successful implementation. The CFIR is intended to be used to collect information (data) from individuals who can influence the implementation outcomes [42, 43]. These frameworks have been selected as they provide a combined focus on implementation outcomes and the contextual factors influencing implementation and to facilitate the development of strategies to support future scale‐up. Thus, our selection of the RE‐AIM and CFIR frameworks is both informed and pragmatic. Pragmatically, the 12‐month project timeframe and the key principles of the project grant, which emphasises partnership and co‐design between industry, end users and universities, influenced the choice.

### 2.3 Setting

This study will be undertaken in two RAC homes in metropolitan South Australia. These facilities are run by a not‐for‐profit organisation and provide three levels of support for people with dementia and complex needs. Site 1 has 115 residents, with eight residents in a Specialist Dementia Care Unit with severe behavioural and psychological symptoms of dementia and three memory support units for residents with dementia (two with nine residents and one with 15 residents). Site 2 has 76 residents with nine residents in a memory support unit.

At each site, person‐centred care is delivered by teams with a skill mix, which includes a clinical care manager, clinical nurse, registered nurse, enroled nurses, personal care workers and lifestyle assistants. Additional staff include the Hotel Services team including kitchen hands, cleaners, administration and maintenance staff, who interact with residents without providing personal care. Nurses are registered with the Australian Health Practitioner Regulation Agency and personal care workers and lifestyle assistants require a minimum of Certificate III Individual Support or similar. The Specialist Dementia Care Unit receives additional support from a nurse consultant employed by a local health network older persons’ mental health service.

### 2.4 Participants

Aged care staff from a range of disciplinary backgrounds (including, managers, educators, nurses and aged care workers), who work at the participating facilities, and key leaders will be invited to participate. Participation is voluntary with no exclusion criteria. Staff can attend the training without having to consent to participate in the research. Residents who have capacity to consent and participate, as assessed according to the Guardianship and Administration Act 1993 (South Australia), and families who provide consent will be recruited.

### 2.5 Description of Safewards

The Safewards programme includes a conceptual model that explains conflict and containment in acute psychiatric settings and a set of 10 interventions to reduce rates of conflict and containment [4, 25]. Conflict refers to patient behaviours that threaten their safety or the safety of others (e.g., violence, self‐harm and absconding), and containment refers to staff actions that aim to prevent events from occurring or to minimise harm (e.g., medication, special observation and physical restraint). The model identifies six originating domains that influence conflict and containment: the staff team, the patient community, patient characteristics, the physical environment, outside the hospital and regulatory frameworks. All domains are relevant to RAC homes. The Safewards model explains the relationship between conflict and containment and highlights opportunities for staff to intervene to prevent conflict and respond with least restrictive practices (through the application of the 10 interventions; Table 1).

**Table 1:** Safewards interventions (from www.safewards.net).

**Intervention**	**Description**
Clear mutual expectations	Co‐producing expectations between staff and consumers to create mutual clarity and consistency.
Soft words	Soft words aimed at showing empathy, kindness and respect to consumers and colleagues.
Talk down	Training staff in de‐escalation techniques to help calm consumers who are distressed and reduce conflict.
Positive words	Staff providing positive information about consumers during the handover, including explanations as to why a consumer may have been distressed.
Bad news mitigation	Actively planning how to share/communicate bad news with consumers.
Knowing each other	Sharing interests and appropriate information between staff, consumers and carers to engage and build relationships.
Calm down methods	Providing alternative methods to help calm consumers who are distressed.
Reassurance	Providing reassurance to consumers following stressful incidents on the unit.
(Discharge messages) Meaningful messages	Messages of positivity to reassure others and promote hope.
Mutual help meeting	Voluntary meetings between staff and consumers to discuss potential improvements to the unit.

### 2.6 Implementation Process

**Figure 1:**
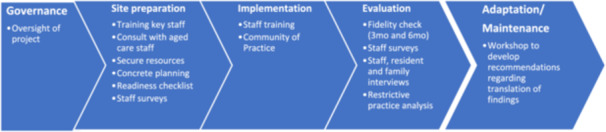
Implementation process.

This project will occur over five stages as detailed in Figure 1.

#### 2.6.1 Governance

A steering group of key stakeholders will be formed to provide oversight and expert advice as the trial progresses. Stakeholders will include Safewards trainers (including a trainer with lived experience), staff from the aged care organisation, consumers (residents and family members) and researchers with expertise in implementation research, Safewards and restrictive practice use.

#### 2.6.2 Site Preparation and Training

This stage includes in‐depth 2‐day training for key staff members at the participating sites (including managers, educators, nurses and aged care workers), identification of staff to lead the range of interventions and co‐development of an implementation plan for each site. Implementation plans/processes (e.g., Safewards interventions readiness checklist) from Safewards UK will inform implementation. Adaptations/tailoring of the Safewards interventions will be discussed at a weekly implementation working group, held with the trainers and research team and key organisational staff (e.g., clinical care managers and the clinical educator; Table 2). An expert in Safewards will provide ongoing mentoring for the project duration via teams. Details of this will be determined by key staff at the RAC home.

**Table 2:** Implementation strategies.

**Implementation strategy**	**Example**
Evaluative and iterative strategies 1. Assess for readiness and identify barriers and facilitators 2. Purposefully re‐examine implementation 3. Develop and implement tools for quality monitoring	Weekly implementation working group identifies barriers/facilitators and reports to Steering Committee. Evaluation process. Adapt and use Safewards Fidelity Checklist.
Develop stakeholder interrelationships 1. Identify and prepare champions 2. Organise implementation teams 3. Obtain formal commitments 4. Capture and share local knowledge 5. Use advisory boards and workgroups 6. Develop academic partnerships	2× champions for each intervention at each site. Champions to meet as a group at each site. Executive sponsorship obtained. Weekly implementation working group with participants from across both sites and implementation of a Community of Practice. Weekly implementation working group. Project occurring in partnership with the University.
Train and educate stakeholders 1. Develop educational materials 2. Conduct educational outreach visits	Facilitators (from the research team) adapting Safewards materials in collaboration with key staff from the aged care organisation. Facilitators (from the research team) and clinical educator from the aged care organisation providing education on each intervention to aged care staff.
Engage consumers 1. Involve residents and family members	Invitation for involvement in steering committee, participation in selected interventions and evaluation.

#### 2.6.3 Implementation Phase

Implementation of Safewards at each site will occur over 12 weeks. The clinical educator from the aged care organisation and two trainers from the project team (one staff with lived experience of caring for a family member with dementia) will provide on‐site training of aged care staff. An online community of practice groups will be established to share learnings and support implementation across sites.

#### 2.6.4 Evaluation Phase

Programme evaluation will examine the impact of Safewards in aged care homes, and identification of successful implementation strategies and adaptations made to the interventions for service fit. Further details of the outcomes evaluation are provided in Table 3.

**Table 3:** Evaluation summary.

**RE‐AIM domains**	**Evaluation aims and outcome measures**
*Reach:* Participation in the intervention (individual level)	*Aim*: To describe the number, proportion and characteristics of staff who are willing to participate in the intervention. *Data collection: Quantitative*—Number of staff who engaged in the training and participant characteristics (age, gender, qualifications and cultural background).
*Effectiveness*: Impact of the intervention (individual and setting level)	*Aim:* To determine the appropriate and feasible effectiveness outcome measures and potential signals of effect. 1. *Resident outcomes. Data collection: Quantitative*—Pre‐ and postquarterly reports of pro‐re‐nata psychotropic medication use, pre‐ and postquarterly reports of incidents of mechanical, physical and environmental restraint. *Qualitative*—Interviews postimplementation to understand the experiences of the intervention. 2. *Family member outcomes. Data collection: Qualitative*—Interviews postimplementation to understand experiences of the intervention. 3. *Staff outcomes. Data collection: Quantitative:* Pre‐ and postsurveys to assess levels of confidence and capacity to support residents with complex needs and use the intervention, pre‐ and postsurveys to assess staff well‐being. *Qualitative*—Focus groups (or interviews) to understand experiences of the intervention.
*Adoption:* Degree of uptake of intervention (individual and setting level)	*Aim:* To describe the number, proportion and representativeness of staff and settings who delivered the intervention. *Data collection: Quantitative*—Setting exclusions/inclusions (% units and reasons); staff exclusions/inclusions (% of staff invited that participate in training); degree of implementation of Safewards (number of interventions implemented). *Qualitative*—Focus groups (or interviews) to understand staff perceptions of barriers and enablers to setting‐ and staff‐level adoption.
*Implementation:* Degree of intervention fidelity achieved (setting level)	*Aim:* To determine the fidelity of delivery of the intervention. *Data collection: Quantitative:* Postimplementation surveys to evaluate staff perceptions of uptake (feasibility and suitability) and adaptations made to the intervention for the setting; Safewards Fidelity Checklist to assess adherence to the Safewards programme (to be conducted 3 months and 6 months after commencement of Safewards).
*Maintenance:* Degree of sustained uptake of intervention	*Aim:* To determine the extent to which the intervention is sustained and embedded in routine practice/policy. *Data collection: Quantitative*—Postimplementation surveys to evaluate staff perceptions of sustainability; Safewards Fidelity Checklist to assess adherence to the Safewards programme (to be conducted 3 months and 6 months after commencement of Safewards). *Qualitative*—Focus groups (or interviews) to understand staff perceptions of barriers and enablers to sustained adoption.
**CFIR domains**	**Evaluation aims and outcome measures**
*Innovation:* Intervention characteristics that influence successful uptake	*Aim*: To identify the key attributes of the intervention that influence the success of implementation (e.g., evidence base, relative advantage, adaptability, complexity, trialability design and cost). *Data collection: Qualitative*—Focus groups and interviews with staff and managers and review of steering committee meeting minutes and research notes.
*Outer setting:* External factors influencing successful implementation	*Aim*: To identify the range of external factors influencing successful implementation (e.g., sociocultural values, policies and legislation, quality benchmarking). *Data collection: Qualitative*—Focus groups and interviews with staff and managers.
*Inner setting:* Aspects of the organisation affecting successful implementation	*Aim:* To identify organisational aspects (e.g., structure, relational connections, function, culture, mission alignment and resources) affecting successful implementation. *Data collection: Qualitative*—Focus groups and interviews with staff and managers.
*Individuals:* Characteristics of individuals that influence successful implementation	*Aim:* To identify participants' roles (staff from a range of disciplines and managers) and personal characteristics that may influence implementation (e.g., need, capability, opportunity and motivation). *Data collection: Qualitative*—Focus groups and interviews with staff and managers.
*Implementation process:* Stakeholders' engagement and decision‐making throughout implementation	*Aim:* To identify stakeholder and participant engagement and decision‐making throughout implementation (stages 1–3 of project design). *Data collection: Qualitative*–Interviews with key stakeholders and managers and review of steering committee meeting minutes and research notes.

#### 2.6.5 Adaption/Maintenance of Safewards

Staff from a range of disciplinary backgrounds and RAC homes, along with residents and family members, will be invited to participate in a workshop to develop recommendations regarding translation and expansion of Safewards in RAC homes. This will include feasible strategies/recommendations for future implementation and maintaining the Safewards programme.

### 2.7 Implementation Strategies

Implementation strategies are the ‘methods or techniques used to enhance the adoption of a clinical programme or practice’ [47, p. 2] Implementation strategies will be guided by resources that are publicly available to support the implementation and evaluation of Safewards detailed on the UK Safewards site (https://www.safewards.net/) and the Victorian Safewards trial site (https://www.health.vic.gov.au/practice-and-service-quality/safewards-victoria). Specific implementation strategies to be used are described in Table 2 and listed according to results from the Expert Recommendations for Implementing Change (ERIC) study [48]. The ERIC study compiled 73 discreet implementation strategies (with definitions) to support the process of implementation itself, including the assessment of effective strategies and reporting of outcomes [48].

### 2.8 Outcomes Evaluation

The RE‐AIM [41] and CFIR [42, 43] frameworks will be applied in combination to evaluate the implementation of Safewards in RAC homes. Both quantitative and qualitative data will be used in the evaluation process. RE‐AIM will be used to describe the individual and setting‐level impact of Safewards (Reach and Effectiveness) and the setting‐level factors that support successful implementation (Adoption, Implementation and Maintenance). Table 3 provides a definition of the RE‐AIM and CFIR domains, and details on how each construct is being applied in the evaluation.

An interview schedule for aged care staff and managers, based on the CFIR domains and constructs that are most relevant to this project, has been developed to elucidate the facilitators and barriers to effective implementation (Appendix S1).

### 2.9 Data Collection and Analysis

Due to practical considerations and budgetary constraints, we will use routinely collected data pertaining to changes in restrictive practice use and staff well‐being. All staff will be invited to participate in staff surveys exploring their confidence to manage residents with challenging behaviours and motivation to change practice. We anticipate data saturation with participant numbers for the interviews and focus groups.

**Quantitative Data**

1. Routinely collected data pertaining to pro re nata (PRN) psychotropic medication use (from the organisation's psychotropic register where nursing staff record PRN psychotropic medication) and inappropriate restrictive practice incidents (as reported via the Serious Incident Reporting Scheme‐SIRS) will be collected by staff from the aged care organisation and forwarded to the research team for analysis. The total number of PRN psychotropic medications used and restrictive practice events on each individual resident will be calculated over three time periods, which are (1) 12 months before the implementation of the Safewards programme; (2) from start to completion of the programme (3 months) and (3) 3‐months post the completion of the programme. The average use of PRN psychotropic medications for each individual resident per time period, and the total number of restrictive practice events per individual resident per period will be calculated. The difference between the medication usage and a number of events before and after the implementation of the programme will be analysed using the analysis of variance method. The Australian Aged Care Quality & Safety Commission govern the SIRS, which aims to identify and prevent abuse and neglect among people receiving aged care. As only ‘inappropriate’ restrictive practice use is reportable to SIRS, and there is a very low reporting rate sector wide (e.g., < 200 reports of inappropriate restrictive practice use across the entire Australian RAC sector in the October–December 2023 quarter), these data are likely to be limited.
2. Surveys (preimplementation and 1 month postimplementation) exploring staff confidence, motivation and uptake of nonrestrictive practices will be forwarded to all aged care staff (*n* = 200). Surveys have been adapted from the Victorian trial (Appendix S2). All data will be de‐identified. Staff will be asked to identify site location and provide a unique identifier to allow for pre‐ and postdata to be combined. Comparisons will be made pre‐ and post‐Safewards implementation. The online platform Qualtrics will be used to collect survey data.
3. Routinely collected data pertaining to resident satisfaction with care and quality of life is routinely collected by the organisation for reporting purposes (https://www.qol-acc.org/). The most recent survey data available before the intervention and at the completion of implementation phase will be used for analysis.
4. Routinely collected data pertaining to staff satisfaction (from a staff satisfaction survey developed by Willis Towers Watson) and staff injury (via the Injury Management platform). All data will be de‐identified by staff working in the aged care organisation. The most recent survey data available before the intervention and at the completion of implementation phase will be used for analysis.
5. The Victoria Safewards fidelity scale (https://www.safewards.net/managers/evaluation-methods) will be adapted for the setting and completed by two members of the research team on completion of the implementation phase and 3 months postimplementation. The fidelity scale reports on the number and quality of interventions that have been implemented.

We will analyse the data based on the available information on PRN psychotropic mediation and restrictive practice events. No imputation method will be applied due to the high possibility of introducing bias. For all the surveys, the incomplete information will be treated with appropriate imputation techniques consistent with the scoring protocol of the respective survey.

**Qualitative Data**

1. In‐depth interviews with up to 12 residents and 12 family members (six from each site) will be conducted approximately 6 months after the commencement of Safewards implementation. Families will be offered to meet face to face in a quiet room in the residential setting, or via telephone to minimise cost and burden We will explore residents' and family members' perspectives and experiences of Safewards.
2. Focus groups (with up to eight participants) will be conducted with aged care staff to gain their views on the implementation of Safewards. Three face‐to‐face focus groups will be conducted at each site. Focus group methodology is best suited to gaining views within a collaborative and problem‐solving approach. We will explore experiences of Safewards, barriers and enablers to implementation and any adaptations made to Safewards interventions and sustainability.
3. In‐depth face‐to‐face interviews with the aged care organisation leaders will be conducted to gain their views on the implementation of Safewards.
4. Steering Committee Meeting Minutes will be reviewed for data pertaining to implementation.

Interviews and focus groups will be conducted by members of the research team and will be audio recorded with permission from participants, to ensure the accuracy of the data capture, and professionally transcribed.

For the qualitative data, a coding reliability approach to thematic analysis will be used to ensure rigour [49]. This will involve the use of a ‘definitive list of codes/themes, a coding label and definition for each code/theme, instructions on how to identify each code/theme, including any exclusions, and examples of each code/theme’ [49, p. 7]. The CFIR codebook will be used to code the data. This includes a description of each domain and sub‐domain and construct definitions to assist coding (https://cfirguide.org/constructs/). Interview and focus group data will be mapped against the CFIR framework domains, with the researchers employing a coding reliability approach to ensure rigour. NVivo 12 will be used to manage data.

### 2.10 Ethical Considerations

Ethics was sought from the Flinders University Human Research Ethics Committee to conduct this study. A waiver for consent was sought for de‐identified data including the following: (1) routinely collected data pertaining to psychotropic medication use and restrictive practice incidents (physical, mechanical and environmental restraint); (2) staff satisfaction surveys and (3) consumer satisfaction and quality of life questionnaires.

Data pertaining to psychotropic medication use will be collected and de‐identified by staff from the aged care organisation via an electronic record and/or the psychotropic register. Restrictive practice incidents, documented on internal incident report forms, will be collected and de‐identified by organisational staff. Anonymous staff and consumer satisfaction and quality of life surveys are routinely collected for reporting purposes.

## 3 Discussion

Safewards is an evidence‐based programme that provides staff with specific interventions that reduce conflict and restrictive practice use (specifically seclusion) in acute mental health care settings. The Safewards programme (model and interventions) has been successfully implemented in a range of mental health care settings internationally but has not yet been tested in RAC. This study will explore the feasibility of Safewards in reducing restrictive practice use in RAC homes. Given the importance of contextual factors affecting implementation outcomes, this pilot study will apply theoretical frameworks (RE‐AIM and CFIR) to gain an in‐depth understanding of the feasibility and adaptations needed for a future trial to determine effectiveness. The following section explores the knowledge areas that this study seeks to develop.

### 3.1 Contribution to the Knowledge of Evidence‐Based Interventions to Reduce Restrictive Practices in RAC Homes

Clinical guidelines prioritise nonpharmacological interventions for people with behavioural and psychological symptoms of dementia [6]. As such, various strategies and interventions have been developed to reduce the use of physical restraints and the overprescribing of psychotropic medication in RAC [18]. Strategies have included education, guidelines, medication reviews, audits, strategies to improve interdisciplinary communication and tips for more personalised care [18]. Although studies that have targeted overprescribing report short‐term improvements, changes are not typically sustained [18].

Overall, there is little evidence on strategies and interventions that have been found to be effective in reducing restrictive practice use in RAC. We suggest that the considerable research on strategies to reduce restrictive practices that has occurred in adult mental health inpatient settings are tested in RAC to assess effectiveness and feasibility. This approach is supported by others, including the Disability Sector. A recent review commissioned by the Australian Royal Commission into Violence, Abuse, Neglect and Exploitation of People with Disability of initiatives to reduce or eliminate the use of restrictive practices acknowledged that most of the evidence on interventions to reduce restrictive practices comes from mental health care settings and encouraged their adoption/trial into the Disability Sector [50]. Cortis et al. [50] identified seven evidence‐based strategies/interventions, which included Safewards. Safewards is broader in its approach than many of the specifically targeted interventions to reduce psychotropic prescribing, identifying six domains which can be the source of conflict and containment (use of restrictive practices) [25]. Adapted for RAC, these domains include the resident community, resident characteristics, regulatory frameworks, the staff team, the physical environment, and outside the care home. By implementing and evaluating Safewards in RAC, this study will contribute to the knowledge about strategies to reduce the range of restrictive practice use (physical, chemical and environmental) in RAC and determine whether adaptations are needed due to contextual differences.

### 3.2 Contribution to the Knowledge Base for Safewards

As highlighted, Safewards was designed to reduce conflict and containment in acute mental health care settings [25], with growing interest in exploring its use in other settings [50]. In Australia, for example, the Victorian Safewards trial included three emergency departments and six general health units (https://www.safercare.vic.gov.au/best-practice-improvement/improvement-projects/mental-health-wellbeing/safewards-victoria-trial) and the ACT Safewards trial included two general medical wards and an older persons mental health unit (https://www.health.act.gov.au/safewards). This study will be conducted in a novel context (RAC), with the variation in services across the two sites (residential home care, memory support units and a specialist dementia care unit) providing an ideal setting for this trial.

Recent systematic reviews of published studies of Safewards have included trials that have been conducted in the United Kingdom, Germany, Poland, Denmark, Canada and Australia [22, 51]. These reviews on the effect of Safewards report overall reductions in conflict and containment in general adult mental health units but highlight the need for further rigorous testing of the model in other settings [22, 24]. In addition to examining changes in restrictive practice use and staff experiences, our will study will include the examination of the experiences of residents and family members of Safewards in RAC. Inclusion of services users has been identified as a key priority when trialling interventions (including Safewards) to reduce restrictive practice use [50, 51], although consumer involvement remains infrequent in aged care implementation research [32].

Although there are acknowledged challenges in implementing complex interventions, only one Safewards study has used implementation science to inform programme evaluation. Fletcher et al. [30] conducted a postevaluation of the barriers and enablers of implementation of Safewards across 18 wards in Victoria using the CFIR framework. More recently, two systematic reviews have applied implementation science to examine the implementation of Safewards and other programmes to reduce coercive practices [33, 52]. Björkdahl et al. [52] synthesised the qualitative research on staff experiences of the barriers and enablers to the implementation of Safewards using the i‐PARIHS framework. Lantta et al. [33] examined the use of models, theories and frameworks used in the implementation of the broad range of programmes designed to reduce coercive practices in mental health settings, which included Safewards, but found only nine papers reporting on eight studies met the inclusion criteria [33]. The authors highlighted the variation in selection and use of theories, models and frameworks across the nine implementation studies. The most frequently reported outcomes were acceptability (7/9 papers), appropriateness (8/9) and sustainability (7/9); penetration was the least reported outcome (1/9) [33].

Our research will extend the evaluation of Safewards to RAC and simultaneously contribute to the knowledge base of implementation science in RAC, where there is limited implementation science‐informed research [32]. The CFIR framework was applied to the Victorian Safewards implementation [37], thus using this framework in our current evaluation will allow for a comparison of findings to determine if there are key components across organisations and sectors that facilitate and/or hinder the successful implementation of Safewards.

### 3.3 Strengths and Limitations of the Study

To our knowledge, this will be the first study to report on the impact and implementation of the Safewards programme into RAC. If this pilot trial demonstrates evidence of positive impacts for residents, families and/or staff, further roll‐out of Safewards across RAC has the potential to positively address the issue of overuse of restrictive practices. Regardless, findings from this study will inform future implementation strategies of novel interventions in RAC. This includes testing the feasibility of data collection to answer the research questions. The limitations include the 12‐month project timeframe and the extensive reform that is currently taking place in the aged care sector resulting in a range of changes in service provision. Although the latter can be viewed as a potential facilitator, there are currently significant demands on RAC to align service provision to the recommendations from the Royal Commission.

## 4 Conclusion

Restrictive practice use is a complex problem requiring sustained attention, co‐production and testing of multicomponent interventions [53]. To promote the rights, freedoms and dignity of people living with complex needs in aged care, approaches that are feasible, effective and sustainable are urgently needed [54]. This project will extend the investigation of Safewards and test the implementation of the model and interventions in two RAC homes. Project findings will provide an in‐depth understanding of the successful implementation processes, the impact of Safewards in reducing restrictive practices and recommendations for wider implementation of Safewards across RAC. The benefits of this project include building the competencies of the aged care workforce and improving the quality of life for people living with dementia and other complex needs in RAC homes.

## Author Contributions

**Suzanne Dawson:** conceptualisation, methodology, funding acquisition, writing–original draft, writing–review and editing, project administration. **Candice Oster:** conceptualisation, methodology, writing–review and editing. **Michael Page:** writing–review and editing, conceptualisation. **Stacey George:** conceptualisation, methodology, supervision, writing–review and editing.

## Ethics Statement

Ethics was sought from the Flinders University Human Research Ethics Committee to conduct this study. A waiver for consent was sought for de‐identified data, which includes the following: (1) routinely collected data pertaining to psychotropic medication use and restrictive practice incidents (physical, mechanical and environmental restraint); (2) staff satisfaction surveys and (3) consumer satisfaction and quality‐of‐life questionnaires. Data pertaining to psychotropic medication use will be collected and de‐identified by staff from the aged care organisation via an electronic record and/or the psychotropic register. Restrictive practice incidents, documented on internal incident report forms, will be collected and de‐identified by organisational staff. Anonymous staff and consumer satisfaction and quality‐of‐life surveys are routinely collected for reporting purposes.

## Supporting information

Supporting information.

Supporting information.

## Data Availability

The authors have nothing to report.
