# Supplementary material for: Exploring the Safewards Programme to Reduce Restrictive Practices in Residential Aged Care: Protocol for a Pilot and Feasibility Study
Source: Health Expect. 2024 Sep 30;27(5):e70037. doi: 10.1111/hex.70037 (PMC11440635; doi:10.1111/hex.70037)
Supplement: Supplementary file 1 — Supporting information. [file HEX-27-e70037-s001.docx]

**Appendix 1. Safewards Implementation**

**Implementation outcomes interview questions**

Thanks so much for speaking with me today. The purpose of this focus group is to see how the implementation of Safewards has gone for you all, and to explore what has and hasn’t worked well. Everything we discuss today will be confidential and you won’t be identified in any reports of publications.

We’ll start with some broad discussion about the Safewards model and interventions, and then drill down to some specifics about why you think things have gone well or not so well for you and others at Aldersgate/Murray Mudge.

**Implementation of Safewards**

Tell me about the uptake of Safewards? *(for senior leadership only)*

- Have you used any Safewards interventions?

If yes,

- How often have you used Safewards interventions?
  - Can you give some examples of when/how it is used?

If no,

- Tell me about why you haven’t used Safewards?
- What has got in the way of using Safewards?

**Intervention characteristics**

- How do the Safewards interventions fit in your setting (Aldersgate/Murray Mudge)? *(prompt- residents, current practice and staff skills)*
  - Can you tell me about any adaptations that were made to the model?
  - Can you tell me about any adaptations that were made to the interventions?

Overall, how well has Safewards been received at Aldersgate/Murray Mudge?

**Outer setting**

- How well does Safewards fit with requirements around mandatory reporting of critical incidents?
  - In what way?
- How well does Safewards fit with relevant Aged Care legislation and policies?
  - In what way? *(for senior leadership only)*

**Inner setting**

- What supports were available to help adopt Safewards?
  - Training
  - Support
- To what extent were these effective in helping you to adopt Safewards interventions?
- How well does Safewards meet the needs of your residents?
  - In what way?
- How do you think Aldersgate’s/Murray Mudges’s culture (values and ways of doing things) affects the uptake of Safewards?
- What motivated you to implement Safewards in your setting? (*for senior leadership only*)

**Characteristics of individual**

- What role have you had in the Safewards trial? *(leadership, trainer, lead for an intervention)*
- What has your motivation been to use Safewards in your workplace?
- How confident are you about being able to use Safewards interventions?
- How confident are your colleagues?

**Process**

- How does Safewards fit with usual work processes at Aldersgate/Murray Mudge?

**Sustainability**

- How likely will Safewards interventions continued to used in your setting in the long term?
- What would assist you (and others) to continue to use Safewards interventions?

**Anything else we haven’t covered**
