# Supplementary material for: Exploring the Safewards Programme to Reduce Restrictive Practices in Residential Aged Care: Protocol for a Pilot and Feasibility Study
Source: Health Expect. 2024 Sep 30;27(5):e70037. doi: 10.1111/hex.70037 (PMC11440635; doi:10.1111/hex.70037)
Supplement: Supplementary file 2 — Supporting information. [file HEX-27-e70037-s002.docx]

Appendix 2. Pre and Post staff confidence/motivation surveys

1. **Pre staff survey**

***Please rate your level of agreement with the following statements:***

**I have the knowledge and skills to support residents who are distressed, agitated or angry**

1. Strongly disagree
2. Disagree
3. Neither agree nor disagree
4. Agree
5. Strongly agree

**I have the confidence to support residents who are distressed, agitated or angry**

1. Strongly disagree
2. Disagree
3. Neither agree nor disagree
4. Agree
5. Strongly agree

**I am willing to learn new ways to support residents who are distressed, agitated or angry**

1. Strongly disagree
2. Disagree
3. Neither agree nor disagree
4. Agree
5. Strongly agree

**I know how to get more help to support residents who are distressed, agitated or angry**

1. Strongly disagree
2. Disagree
3. Neither agree nor disagree
4. Agree
5. Strongly agree
6. **Post staff survey**

***Please rate your level of agreement with the following statements:***

**I have the knowledge and skills to** **support residents who are distressed, agitated or angry**

1. Strongly disagree
2. Disagree
3. Neither agree nor disagree
4. Agree
5. Strongly agree

**I have the confidence to support residents who are distressed, agitated or angry**

1. Strongly disagree
2. Disagree
3. Neither agree nor disagree
4. Agree
5. Strongly agree

**I am willing to learn new ways to support** **residents who are distressed, agitated or angry**

1. Strongly disagree
2. Disagree
3. Neither agree nor disagree
4. Agree
5. Strongly agree

**I know how to get more help to support residents who are distressed, agitated or angry**

1. Strongly disagree
2. Disagree
3. Neither agree nor disagree
4. Agree
5. Strongly agree

The following questions are about the Safewards model and interventions.

**Experience of the Safewards model and 10 interventions**

Please indicate which of the 10 Safewards interventions you remember being used at your facility (please tick only one response per line)


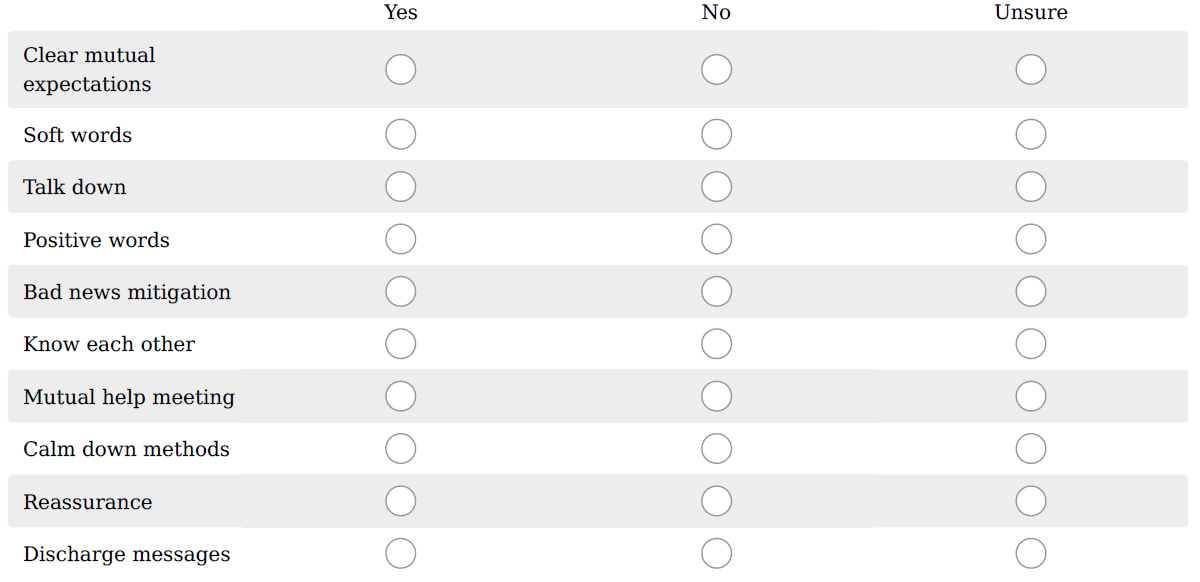


Please rate how suitable the Safewards model and 10 interventions were for your facility.


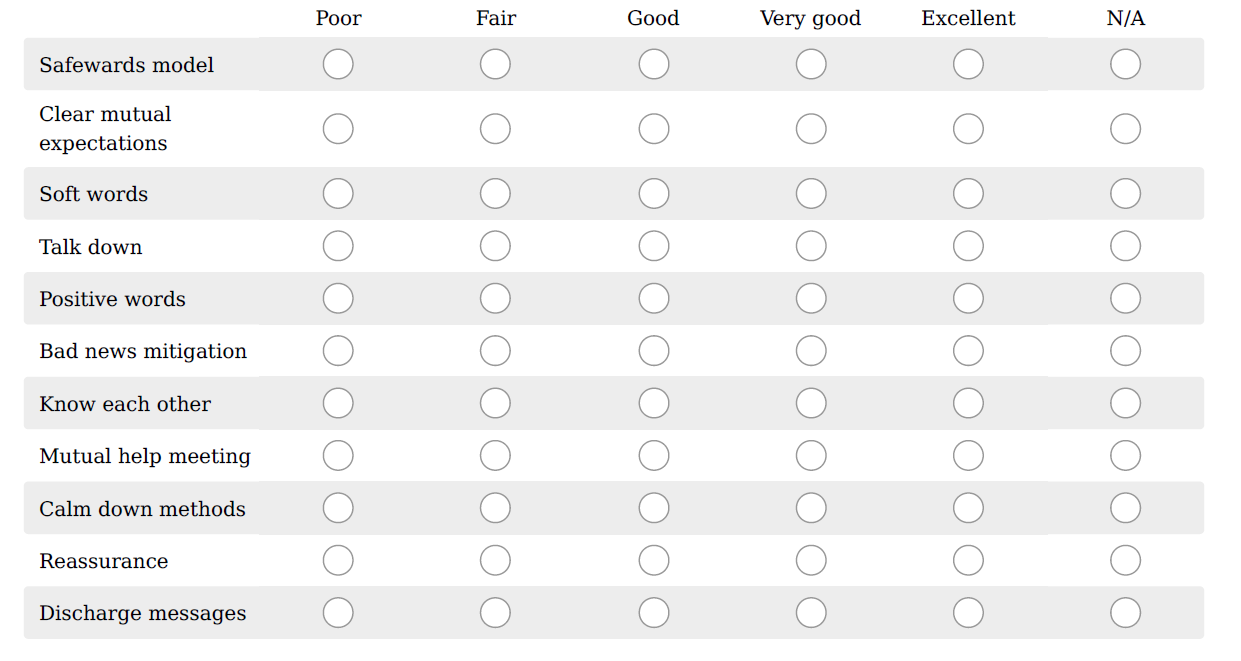


If you have rated the Safewards model or any of the 10 interventions as ‘poor’ can you briefly describe why the model/specific interventions were not suitable for your facility?


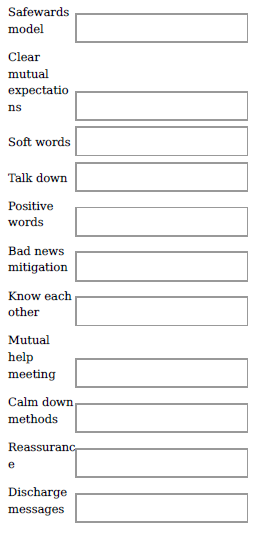


If you have rated the Safewards model or any of the 10 interventions as ‘excellent’ can you briefly describe why the model/specific interventions were suitable for your facility?


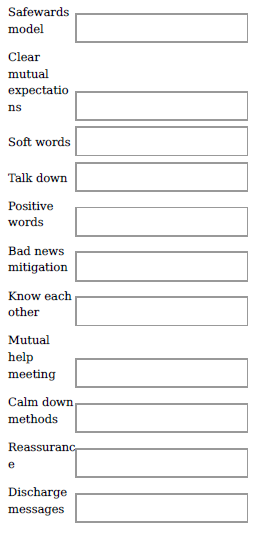


**Use of Safewards**

**Please rate how often** you use each of the 10 Safewards interventions (please tick only one response per line)


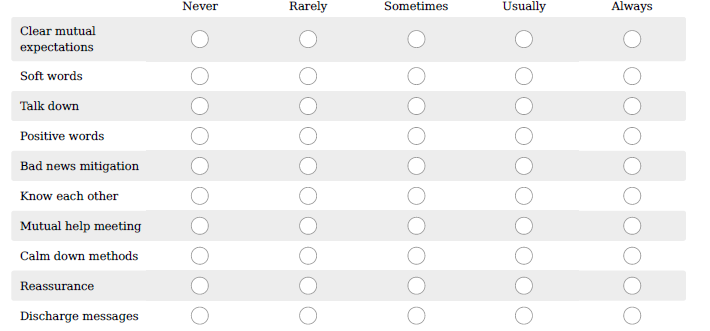


Did using Safewards require you to change your approach to **your work**?

If you answered yes to Question 15, please provide an example

Yes

No

If you answered yes, please provide an example

Did implementing Safewards in your facility require changes to be made to the way your facility worked?

Yes

No

If you answered yes, please provide an example

**Impact of Safewards**

Has Safewards helped to reduce the number of incidents in your facility?


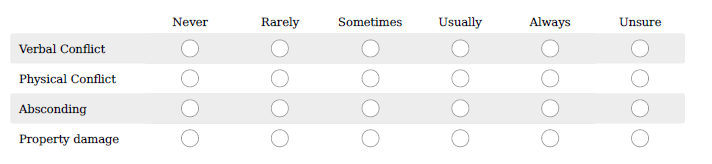


**Sustainability of Safewards**

How likely is it that the Safewards model and 10 interventions will still be in place within your service in 12 months’ time?


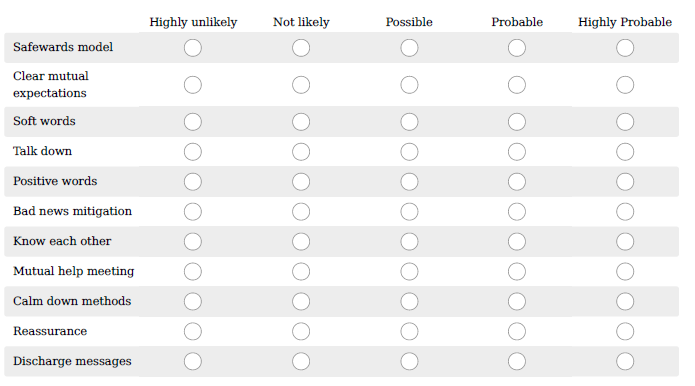


20. What are the reasons that you think Safewards will (or will not) be in place in 12 months time?
